# Abdominal infections in the intensive care unit: characteristics, treatment and determinants of outcome

**DOI:** 10.1186/1471-2334-14-420

**Published:** 2014-07-29

**Authors:** Jan De Waele, Jeffrey Lipman, Yasser Sakr, John C Marshall, Philippe Vanhems, Casiano Barrera Groba, Marc Leone, Jean-Louis Vincent, for the EPIC II Investigators

**Affiliations:** Department of Critical Care Medicine, Ghent University Hospital, De Pintelaan 185, 9000 Gent, Belgium; Department of Intensive Care Medicine, Royal Brisbane and Women’s Hospital, The University of Queensland, Butterfield St, Herston, QLD 4029 Australia; Department of Anesthesiology and Intensive Care, Friedrich-Schiller-University, Bachstrasse 18, Jena, D-07743 Germany; Department of Surgery, Interdepartmental Division of Critical Care Medicine, University of Toronto, St. Michael's Hospital, 4th Floor Bond Wing, Room 4-007, 30 Bond Street, Toronto, Ontario M5B 1 W8 Canada; Pôle Santé, Recherche, Risques et Vigilances, Groupement Hospitalier Edouard Herriot, Service d’Hygiène, Epidémiologie et Prévention, Université Lyon 1, Batiment 1, 5 place d'Arsonval, 69437 Lyon cedex 03, France; Department of Intensive Care, Brighton and Sussex University Hospitals NHS Trust, Royal Sussex County Hospital, Eastern Road, East Sussex, Brighton BN2 5BE UK; Department of Intensive Care and Anesthesiology, Hôpital Nord, AP-HM Unité de Recherche en Maladies Infectieuses et Transmissibles (URMITE), Aix-Marseille University, Chemin des Bourrely, 13015 Marseille, France; Department of Intensive Care, Erasme Hospital, Université Libre de Bruxelles, route de Lennik 808, 1070 Brussels, Belgium

**Keywords:** Abdominal infection, Abscess, Peritonitis, Severe sepsis, Critical care, Antibiotic therapy, Microbiology

## Abstract

**Background:**

Abdominal infections are frequent causes of sepsis and septic shock in the intensive care unit (ICU) and are associated with adverse outcomes. We analyzed the characteristics, treatments and outcome of ICU patients with abdominal infections using data extracted from a one-day point prevalence study, the Extended Prevalence of Infection in the ICU (EPIC) II.

**Methods:**

EPIC II included 13,796 adult patients from 1,265 ICUs in 75 countries. Infection was defined using the International Sepsis Forum criteria. Microbiological analyses were performed locally. Participating ICUs provided patient follow-up until hospital discharge or for 60 days.

**Results:**

Of the 7,087 infected patients, 1,392 (19.6%) had an abdominal infection on the study day (60% male, mean age 62 ± 16 years, SAPS II score 39 ± 16, SOFA score 7.6 ± 4.6). Microbiological cultures were positive in 931 (67%) patients, most commonly Gram-negative bacteria (48.0%). Antibiotics were administered to 1366 (98.1%) patients. Patients who had been in the ICU for ≤2 days prior to the study day had more *Escherichia coli*, methicillin-sensitive *Staphylococcus aureus* and anaerobic isolates, and fewer enterococci than patients who had been in the ICU longer*.* ICU and hospital mortality rates were 29.4% and 36.3%, respectively. ICU mortality was higher in patients with abdominal infections than in those with other infections (29.4% vs. 24.4%, p < 0.001). In multivariable analysis, hematological malignancy, mechanical ventilation, cirrhosis, need for renal replacement therapy and SAPS II score were independently associated with increased mortality.

**Conclusions:**

The characteristics, microbiology and antibiotic treatment of abdominal infections in critically ill patients are diverse. Mortality in patients with isolated abdominal infections was higher than in those who had other infections.

**Electronic supplementary material:**

The online version of this article (doi:10.1186/1471-2334-14-420) contains supplementary material, which is available to authorized users.

## Background

Abdominal infection is a common indication for admission to the intensive care unit (ICU) and the abdomen is the second most common site of invasive infection among critically ill patients in epidemiological [1–3] and therapeutic [4] studies. Abdominal infections are more often associated with septic shock and acute kidney injury than are infections in other sites [5, 6]. The spectrum of disease and severity is broad and management of these infections is challenging [7–9].

Multicenter data on the clinical features and microbiology of abdominal infections in the critically ill are rare, and often limited to a single region or country. In recent years, an increase in abdominal infections due to nosocomial and resistant organisms has been reported [10–14], but large-scale data are lacking. Although outcomes may have improved over the years [15], abdominal infections still carry a significant mortality risk. Isolation of nosocomial microorganisms [16], enterococci [17] or fungi [18, 19] is often cited as contributing to mortality, but the extent to which these organisms contribute to that risk is unknown. The role of comorbidities as well as demographic characteristics has also not been studied on a large scale. Prolonged stay in a critical care environment may be associated with changes in microbiology, thus affecting empirical antibiotic treatment, yet recent guidelines do not include length of stay as a potential surrogate marker for the presence of nosocomial or less susceptible microorganisms [20].

The Extended Prevalence of Infection in the ICU (EPIC) II study was a large one-day point-prevalence study of infections in the ICU. The study showed that half of all ICU patients were infected on the study day and 71% were being treated with antibiotics [1]. We used the data collected in the EPIC II study to (1) analyze the characteristics of abdominal infections (patient characteristics, micro-organisms) as well as the antibiotics used to treat these infections; (2) explore the differences in microbiology according to the length of stay in the ICU; and (3) identify clinical and microbiological factors associated with mortality.

## Methods

The EPIC II study was performed on May 8, 2007. Demographic, physiological, bacteriological and therapeutic data were collected from 13,796 adult (>18 years) patients in 1,265 participating ICUs from 75 countries (see Appendix for list of participating centers) on the study day as previously described [1]. The EPIC II study was approved by the Erasme Hospital ethics committee. Local ethical committee approval at each participating center was expedited or waived because of the purely observational nature of the study. Infection was defined according to the criteria of the International Sepsis Forum (ISF) [21] and classified by the attending physician. Microbiological analyses were performed locally. Participating ICUs were asked to provide patient follow-up until hospital discharge or for 60 days.

For the purposes of this study, we analyzed data from the patients who were diagnosed with an intra-abdominal infection.

### Statistics

Statistical analyses were performed using PASW Statistics 18 for windows (SPSS Inc., Chicago, USA). Data are presented as mean (±standard deviation [SD]), median (interquartile range [IQR]), or number (%) as appropriate. To identify factors associated with mortality, a multivariable logistic regression model (single step, forced entry) was constructed using variables for which the P-value was <0.1 in univariable analysis. Goodness of fit was assessed by the Hosmer-Lemeshow statistic. All tests were two-tailed, and a P < 0.05 was considered statistically significant.

## Results

Of the 7,087 infected patients, 1,392 (19.6%) were diagnosed as having an abdominal infection on the study day (Table 1). Cancer and chronic obstructive pulmonary disease (COPD) were the most frequent comorbidities. The majority of the patients (885 [63.7%]) had undergone emergency surgery. Other concomitant infections were frequently present, with respiratory infections and bloodstream infections occurring in 26.8% and 11.6% of the patients, respectively (Additional file 1: Table S1).

**Table 1:** **Patient characteristics**

**Age, mean ± SD, year**	**62 ± 16**
**Male, n (%)**	**831 (60)**
**SAPS II score, mean ± SD**	**38.9 ± 16.4**
**SOFA score, mean ± SD**	**7.6 ± 4.6**
**Length of ICU stay before May 8, median (IQR), days**	**6 (1–15)**
**Type of admission, n (%)**	
Surgical - emergency	**885 (63.7)**
Medical	**260 (18.7)**
Surgical - elective	**198 (14.2)**
Trauma	**47 (3.4)**
**Admission source, n (%)**	
OR/recovery room	**488 (35.3)**
Hospital floor	**465 (33.6)**
ER/ambulance	**199 (14.4)**
Other hospital	**194 (14.0)**
Other	**36 (2.6)**
**Comorbidities, n (%)**	
Cancer	**321 (23.1)**
COPD	**225 (16.2)**
Chronic renal failure	**140 (10.1)**
Insulin dependent diabetes mellitus	**131 (9.4)**
Heart failure (NYHA III-IV)	**107 (7.7)**
Cirrhosis	**79 (5.7)**
Hematological cancer	**24 (1.7)**
HIV	**12 (0.9)**
**Organ support on the study day**	
Mechanical ventilation	**863 (62.0)**
Renal replacement therapy	**220 (15.8)**
**Outcome measures**	
ICU LOS, median (IQR), days	**16 (6–34)**
Hospital LOS, median (IQR), days	**30 (14–59)**
ICU mortality, n (%)	**382 (29.4)**
Hospital mortality, n (%)	**472 (36.3)**

SAPS II = Simplified Acute Physiology Score II; SOFA = Sequential Organ Failure Assessment; HIV = Human Immunodeficiency Virus; NYHA III-IV = New York Heart Association class III-IV.

Microbiological data were available for 931 patients (67%), with a total of 1,289 microorganisms isolated (Table 2). Polymicrobial infections were present in 40.1% of the patients. *Escherichia coli* was isolated most frequently, with *Pseudomonas* spp. and *Klebsiella* spp. ranking second and third among the Gram-negative isolates. *Enterococcus* was the most prevalent Gram-positive isolate. Antibiotic resistance was relatively rare: ampicillin-resistant enterococci were isolated in 70 patients (7.5%), methicillin-resistant staphylococci in 59 patients (6.3%). *Candida* species were isolated in 156 patients (16.8%), 75.6% of these isolates were *Candida albicans*.

**Table 2:** **A total of 1289 micro-organisms were recovered from the 931 patients with abdominal infections and positive cultures**

	n (%)
**Gram-negative bacteria**	**619 (48.0%)**
*Escherichia coli*	211
*Pseudomonas aeruginosa*	86
*Klebsiella* spp.	85
*Enterobacter* spp.	77
*Proteus* spp.	47
*Acinetobacter* spp.	35
*Stenotrophomonas maltophilia*	17
*Citrobacter* spp.	13
*Bacillus*	13
Enterobacteria, other	9
*Campylobacter* spp.	7
*Salmonella* spp.	7
*Serratia* spp.	6
*Pseudomonas*, other than *P aeruginosa*	4
*Haemophilus* spp.	2
**Gram-positive bacteria**	**366 (28.4%)**
Enterococci, ampicillin-sensitive	122
Enterococci, ampicillin-resistant	70
Methicillin-resistant *Staphylococcus aureus* (MRSA)	34
Methicillin-sensitive coagulase-negative staphylococci	27
*Streptococcus*, other than group A, B, C and D	31
Methicillin-resistant coagulase-negative staphylococci	25
Methicillin-sensitive *S. aureus*	21
Group A, B, C, G *Streptococcus*	15
Gram-positive cocci, other	8
Gram-positive bacilli, other	8
*Streptococcus pneumoniae*	5
**Anaerobes**	**146 (11.3%)**
*Clostridium*	94
*Bacteroides*	29
Anaerobes, other	16
Anaerobic cocci	7
**Fungi**	**130 (10.1%)**
*Candida albicans*	118
*Candida* non-albicans	38
Fungi, other	5
*Aspergillus* spp.	2
**Viruses**	**12 (0.9%)**
**Other**	**16 (1.2%)**

Almost all the patients with abdominal infections (98.1%) were receiving antibiotics: penicillins and other beta-lactam antibiotics (excluding cephalosporins) were used most frequently (38.6% and 34.4% of the patients, respectively) (Additional file 1: Table S2); 29.4% of the patients were receiving antifungal agents.

ICU (median 16 [IQR 6–34] days vs. 17 [7–34] days, P = 0.07) and hospital (30 [14–59] days vs. 29 [14–56] days, P = 0.68) lengths of stay (LOS) were similar in patients with abdominal infections and those with other infections. Overall ICU and hospital mortality rates were 29.4% and 36.3%, respectively (Table 1). Mortality rates were higher in patients who had abdominal infections than in patients from the EPIC II cohort who had other infections (ICU mortality 29.4% vs. 24.4%, P < 0.001, and hospital mortality 36.3% vs. 32.3%, P = 0.005). ICU and hospital mortality rates in non-infected patients in the EPIC-II cohort were 10.7% and 14.8%, respectively.

Non-survivors were older, had higher SAPS II and SOFA scores on the study day, and were more likely to have cirrhosis, heart failure, or hematological cancer. They were also more likely to be receiving mechanical ventilation or renal replacement than survivors (Table 3). Survivors and non-survivors had similar patterns of infecting organisms, except for *P. aeruginosa* and *Stenotrophomonas maltophilia*, which were isolated more frequently in non-survivors than in survivors (Additional file 1: Table S3). In multivariable analysis, hematological cancer, mechanical ventilation, cirrhosis, renal replacement therapy and SAPS II score on the study day were independently associated with increased mortality (Table 4).

**Table 3:** **Characteristics of survivors and non-survivors**

	Survivors (n = 917)	Non-survivors (n = 382)	p
Age, mean ± ± SD	61.6 ± 16.5	65.2 ± 14.9	<0.001
Male, n (%)	546 (59.7)	223 (58.5)	0.7
**Severity score on study day**			
SAPS II, mean ± SD	34.2 ± 13.5	50.1 ± 17	<0.001
SOFA, mean ± SD	6.2 ± 3.8	10.5 ± 4.6	<0.001
ICU stay before May 8, median (IQR)	5 (1–13)	8 (1–18)	<0.001
**Type of admission, n (%)**			0.58
Surgical/elective	124 (13.5)	62 (16.3)	
Medical	179 (19.5)	69 (18.2)	
Surgical/emergency	580 (63.2)	237 (62.4)	
Trauma	34 (3.7)	12 (3.2)	
**Admission source, n (%)**			0.12
OR/recovery room	349 (38.2)	120 (31.7)	
Hospital floor	290 (31.8)	141 (37.2)	
ER/ambulance	135 (14.8)	50 (13.2)	
Other hospital	116 (12.7)	57 (15)	
Other	23 (2.5)	11 (2.9)	
**Comorbidities, n (%)**			
COPD	149 (16.2)	62 (16.2)	0.99
Cancer	203 (22.1)	97 (25.4)	0.20
Heart failure (NYHA III-IV)	62 (6.8)	40 (10.5)	0.02
Insulin dependent diabetes mellitus	87 (9.5)	37 (9.7)	0.91
Chronic renal failure	83 (9.1)	44 (11.5)	0.17
Cirrhosis	39 (4.3)	33 (8.6)	<0.01
Hematological cancer	9 (1.0)	14 (3.7)	<0.001
HIV	6 (0.7)	6 (1.6)	0.12
**Treatment on the study day, n (%)**			
Mechanical ventilation	534 (58.2)	329 (86.4)	<0.001
RRT (hemodialysis or hemofiltration)	110 (12.0)	110 (28.9)	<0.001
**Outcome**			
ICU LOS, median (IQR)	14 (5–32)	18 (9–38)	<0.001
**Other sites of infection, n (%)**			
Respiratory	221 (24.1)	120 (31.4)	<0.01
Blood stream	97 (10.6)	56 (14.7)	0.04
Renal/urinary tract	53 (5.8)	33 (8.6)	0.06
Skin	29 (3.2)	23 (6)	0.02
Catheter-related	28 (3.1)	21 (5.5)	0.04
CNS	1 (0.1)	0 (0)	0.52
Others	19 (2.1)	16 (4.2)	0.03

SAPS II = Simplified Acute Physiology Score II; SOFA = Sequential Organ Failure Assessment; HIV = Human Immunodeficiency Virus; RRT = renal replacement therapy; NYHA III-IV = New York Heart Association class III-IV.

**Table 4:** **Multivariable analysis with ICU mortality as the dependent variable**

	Odds ratio	(95% CI)	p-value
Hematological cancer	4.04	(1.47-11.11)	<0.01
Mechanical ventilation	2.97	(2.03-4.35)	<0.001
Cirrhosis	2.35	(1.29-4.30)	<0.01
RRT (hemodialysis or hemofiltration)	1.51	(1.03-2.21)	0.04
SAPS II score on the study day (per point)	1.06	(1.05-1.07)	<0.001

Legend. Adjusted for hospital, organizational factors and for geographic region. CI = confidence interval; SAPS II = Simplified Acute Physiology Score II; RRT = renal replacement therapy. Hosmer-Lemeshow goodness of fit: chi2 = 5.315 with 8 df, p-value = 0.723; the c-statistic 0.82 (95%CI:0.80-0.85), p-value < 0.0001.

In patients who had been in the ICU for 2 days or less prior to the study day, there were more *E. coli*, methicillin-sensitive *S. aureus* and anaerobic isolates and fewer enterococci than in patients who had been in the ICU for a longer period of time; there was also a trend towards fewer *P. aeruginosa*, *Citrobacter* spp. and *C. albicans* isolates (Table 5).

**Table 5:** **Microbiology and antibiotic use in patients who had been admitted for 2 days or less vs. more than 2 days on the study day**

	LOS ≤2d (n = 492)	LOS >2d (n = 899)	P
Microorganisms: Positive isolates	260 (53)	669 (74.4)	<0.001
**Gram-positive bacteria**			
Methicillin-sensitive *S aureus*	10 (3.8)	11 (1.6)	0.04
Methicillin-resistant *Staphylococcus aureus*	7 (2.7)	27 (4)	0.33
Methicillin-sensitive coagulase-negative Staphylococci	4 (1.5)	23 (3.4)	0.12
Methicillin-resistant coagulase-negative staphylococci	5 (1.9)	20 (3.0)	0.37
Enterococci, ampicillin-sensitive	22 (8.5)	100 (14.9)	<0.01
Group A, B, C, G *Streptococcus*	4 (1.5)	10 (1.5)	0.96
*Streptococcus pneumoniae*	1 (0.4)	4 (0.6)	0.69
*Streptococcus*, other than group A, B, C and D	11 (4.2)	20 (3)	0.34
Gram-positive cocci, other	2 (0.8)	6 (0.9)	0.85
Gram-positive bacilli, other	2 (0.8)	6 (0.9)	0.85
Enterococci, ampicillin-resistant	23 (8.8)	47 (7.0)	0.35
**Gram-negative bacteria**			
*Escherichia coli*	74 (28.5)	137 (20.5)	<0.01
*Enterobacter* spp.	22 (8.5)	55 (8.2)	0.91
*Klebsiella* spp.	21 (8.1)	64 (9.6)	0.48
*Proteus* spp.	13 (5.0)	34 (5.1)	0.96
*Salmonella* spp.	2 (0.8)	5 (0.7)	0.97
*Serratia* spp.	2 (0.8)	3 (0.4)	0.55
*Citrobacter* spp.	1 (0.4)	12 (1.8)	0.10
*Pseudomonas aeruginosa*	17 (6.5)	69 (10.3)	0.08
*Pseudomonas*, other than *P aeruginosa*	0 (0.0)	4 (0.6)	0.21
*Stenotrophomonas maltophilia*	5 (1.9)	12 (1.8)	0.90
*Acinetobacter* spp.	8 (3.1)	27 (4)	0.49
*Campylobacter* spp.	4 (1.5)	3 (0.4)	0.09
*Haemophilus* spp.	0 (0.0)	2 (0.3)	0.38
Enterobacteria, other	4 (1.5)	5 (0.7)	0.27
*Bacillus*	3 (1.2)	10 (1.5)	0.69
**Anaerobes**			
*Clostridium*	21 (8.1)	72 (10.8)	0.22
Anaerobic cocci	2 (0.8)	5 (0.7)	0.97
*Bacteroides*	9 (3.5)	20 (3.0)	0.71
Anaerobes, other	9 (3.5)	6 (0.9)	<0.01
Mycobacteria	1 (0.4)	1 (0.1)	0.49
**Fungi**			
Candida albicans	29 (11.2)	89 (13.3)	0.38
Candida non-albicans	6 (2.3)	32 (4.8)	0.09
Aspergillus	0 (0.0)	2 (0.3)	0.38
Fungi, other	1 (0.4)	4 (0.6)	0.69
**Antibiotic use**			
Cephalosporins	115 (23.4)	145 (16.1)	<0.001
Penicillins	191 (38.9)	290 (32.3)	0.01
Other beta lactams	116 (23.6)	331 (36.8)	<0.001
Aminoglycosides	64 (13.0)	114 (12.7)	0.85
Quinolones	67 (13.6)	131 (14.6)	0.64
Glycopeptides	78 (15.9)	252 (28)	<0.001
Macrolides	7 (1.4)	23 (2.6)	0.17
Other antibiotics	194 (39.5)	355 (39.5)	0.99
Antifungals	80 (16.3)	266 (29.6)	<0.001

## Discussion

This study is one of the first to look at abdominal infections in critically ill patients from a global perspective. The results show that abdominal infections are associated with significant mortality rates and that concomitant infections are frequent. Microbiology patterns and antibiotic treatments were diverse in this group of patients, and pathogens were different in patients who had been in the ICU for a longer period of time than in those more recently admitted. The severity of disease and presence of comorbidities determined outcome in these patients.

Mortality in patients who had abdominal infections was significantly higher than in patients who had other infections (most of which were respiratory infections), which was not found in previous studies. In an analysis of patients from the Sepsis Occurrence in Acutely Ill Patients (SOAP) study, Volakli et al. reported no differences in mortality rates among patients with abdominal infections and those with respiratory infections [6]. The higher mortality rate in patients with abdominal infections in our study may be explained by a number of differences between abdominal and other infections. First, timely source control is particularly important in the management of abdominal infections [22], and the method by which source control is obtained may influence outcomes [23]. Failed source control is often difficult to identify and can be a cause of persistent infection. In addition, abdominal infections are typically polymicrobial and often associated with resistant organisms; in the current study, non-survivors more frequently had *P. aeruginosa* and *Stenotrophomonas maltophilia* as pathogens. Finally, the large number of concomitant infections may also have affected outcomes.

As expected, comorbidities, such as cirrhosis and hematological cancer, were associated with increased mortality, as was found in the EPIC II patients in general [1]. Most notably, the impact of cirrhosis was considerable with a 2.3-fold increase in the risk of death. A recent analysis of the Project Impact database also showed that cirrhosis was independently associated with an increased risk of 30-day mortality [24].

The microbiology patterns were diverse, and quite different from those in the overall EPIC II study population, in which staphylococci were isolated most frequently, and Gram-positive and Gram-negative organisms were equally present [1]. In the patients with abdominal infections, the picture was substantially different: Gram-negative bacteria were isolated almost twice as frequently as Gram-positive bacteria, with *E. coli* being the most prevalent pathogen; typical nosocomial microorganisms, such as *P. aeruginosa* and *Enterobacter* spp. were also frequently isolated. Indeed, *P. aeruginosa* was the second most frequently isolated Gram-negative microorganism, which may in part be due to the design of the study, but the percentages are comparable to other studies in this field [25]. Among the Gram-positive microorganisms, enterococci were most prevalent, whereas staphylococci were uncommon. Furthermore, there were important differences in microbiology between survivors and non-survivors, with *P. aeruginosa* and *Stenotrophomonas maltophilia* isolated twice and four times more often, respectively, in non-survivors than in survivors. These pathogens may have a greater degree of pathogenicity (although *Stenotrophomonas* is generally not considered to be a major pathogen), or may be more difficult to treat. Detailed data regarding antibiotic resistance, including information regarding resistance to specific antibiotics, were not collected so we are unable to comment further on these aspects.

The findings in this study suggest that physicians around the world seem to comply with international guidelines in this field as most patients receive broad-spectrum antibiotics, often in combination with agents aimed at fungi or even at resistant Gram-positive microorganisms. We also found that patients who had stayed in the ICU for 2 days or less on the study day had different characteristics to those who had been longer on the ICU. Microbiological isolates and antibiotic treatments were remarkably different between these groups with fewer carbapenems, glycopeptides and antifungals used in patients with shorter stays. Current guidelines for the selection of antibiotic therapy in critically ill patients do not mention length of stay in the hospital as a consideration for empirical treatment in patients with high-severity non-nosocomial infections. Nevertheless, this group represents a category of patients, presumably with community-acquired infection, who could potentially be treated with narrower spectrum antibiotics when local ecology allows. This hypothesis warrants further evaluation.

Fungal infections have received considerable attention in the last decade. Although the debate continues as to whether fungi are relevant in community-acquired disease, the situation is different in nosocomial infections and in severely ill patients [20]. *Candida* isolation has been identified as an independent predictor of mortality in some studies [19, 26, 27], which has triggered widespread use of empiric antifungal coverage with fluconazole in this setting. In the current study, fungi were isolated in approximately 1 in 6 patients, but antifungal therapy was administered to almost 30% of the patients, reflecting the high use of antifungal prophylaxis in this group. Fungi were found more often in patients who had been in the ICU for more than 2 days, but were not linked to mortality in the current study. Identifying which patients are at risk of fungal infection and may benefit from preemptive antifungal therapy remains a challenge; length of stay in the hospital and other risk factors for fungal infection, such as upper gastro-intestinal tract perforation and previous antibiotic exposure [28], should be considered before initiating antifungal therapy. The prevalence of *Candida* non-albicans isolates was lower than frequently reported in invasive candidiasis studies or other studies in patients with *Candida* peritonitis. For example, Montravers et al. reported that only 58% of patients with *Candida* peritonitis had *C. albicans* isolated from intraoperative cultures [18]. In patients with invasive candidiasis, a systematic review by Andes et al. indicated that just 44% of the isolates were *C. albicans*[29]; patients with *Candida* peritonitis accounted for only 1% of the patients in this review, however. It is not clear whether the infecting *Candida* species or its susceptibility plays a major role in determining outcome [18].

This study has a number of limitations. Because the study was not primarily focused on abdominal infections, the exact source and extent of infection were not recorded and the efficacy of source control and appropriateness of antimicrobial therapy could not be evaluated. The rate of superinfection and/or tertiary peritonitis could not be assessed. Data on the community-acquired versus nosocomial nature of infections were also not available and we, therefore, used the length of stay as a surrogate marker, but acknowledge that this has its limitations. Finally, as in all point-prevalence studies, patients who are admitted for a long period of time may skew the findings with more data collected on those who stay in the ICU for a longer period of time.

## Conclusion

In conclusion, this study found that abdominal infections were present in about one fifth of ICU patients on the study day and concomitant infections were common. Microbiology patterns and choice of antibiotic therapy were diverse and differed in patients who had stayed in the ICU for 2 days or less compared to those with longer stays. Abdominal infections carry a poor prognosis, with higher mortality rates than in patients with infections from other sources. Disease severity, need for organ support and presence of comorbidities were independently associated with mortality in our cohort.

## Appendix: List of participating centers by country, alphabetically

*Andorra:* Hospital Nostra Senyora de Meritxell (A Margarit); *Argentina:* Centro de Educación Médica E Investigaciones Clínicas (R Valentini); Clinica de Especialidades Villa Maria (AJ Zazu); Clínica Modelo de Morón (C Bevilacqua); Clinica Y Maternidad Suizo (M Curone); CMIC (R Rabuffetti); Hospital Aleman (P Comignani); Hospital Argerich (M Torres Boden); Hospital Britanico (F Chertcoff); Hospital Central de San Isidro (G Cardonatti); Hospital de Niños Dr. Héctor Quintana (F Adén); Hospital del Niño Jesús (L Marcos); Hospital Dr Pedro Ecay (M Dónofrio); Hospital Español de Mendoza (R Fernández); Hospital Español Medical Plaza (R Lamberghini); Hospital Internacional General de Agudos "José de San Matín" (S Balasini); Hospital Interzonal Dr. O. Alende (J Teves); Hospital Italiano de Buenos Aires (M Las Heras, J Sinner); Hospital Juan A. Fernández (D Ceraso); Hospital Municipal de Chivilcoy (D Curcio); Hospital Profesor Alejandro Posadas (L Aguilar); Hospital Provincial de Rosario (C Weller); Hospital Provincial del Centenario (L Cardonnet); Hospital Regional Rio Gallegos (R Santa Cruz); Hospital Regional Ushuaia (E Manrique); Hospital Universitario Austral (D Bernardez, T Iolster); Hospital Universitario Universidad Abierta Interamericana (G Chiappero); Instituto Privado del Quemado Med-Inter (D Curcio); Nuevo Hospital El Milagro (P Ramos); Ramos Mejia Hospital (J Vergara); Sanatorio Agote (I Moine); Sanatorio de la Trinidad Mitre (S Ilutovich); Sanatorio de Los Arcos (G Jannello); Sanatorio Dupuytren (M Waschbusch); Sanatorio Frangioli de Salud 2000 Srl (G Rios Picaza); Sanatorio Mater Dei (A Raimondi); Sanatorio Otamendi Y Miroli (M Miriam); Sanatorio Parque (L Carlos); Sanatorio San José (D Curcio); *Armenia:* Centro Gallego de Buenos Aires (M Caridi); *Australia:* Alfred Hospital (T Leong); Barwon Health (N Orford); Blacktown Hospital (G Reece); Box Hill Hospital (D Ernest); Cabrini Hospital (F Hawker); Concord Repatriation General Hospital (J Tan); Epworth Eastern Private Hospital (C Giannellis); Epworth Hospital Richmond (B Ihle); Flinders Medical Centre (A Bersten); Frankston Hospital (J McInnes); Gold Coast Hospital (M Tallott); John Hunter Hospital (B Mcfadyen); Joondalup Health Campus (J Vibert); Liverpool Hospital, Sydney South West Area Health Service (M Parr); Logan Hospital (K Tran); Mater Health Services (J Sutton); Mount Hospital (S Webb); Nambour General Hospital (N Groves); Nepean Hospital, NSW (L Cole); Prince Charles Hospital (D Long); Prince of Wales Hospital (F Bass); Princess Margaret Hospital For Children (S Erickson); Royal Brisbane and Womens' Hospital (J Lipman); Royal Children’s Hospital, Brisbane (D Long); Royal Children’s Hospital, Melbourne (C Delzoppo); Royal Darwin Hospital (J Thomas); Royal Perth Hospital (G Dobb); Royal Prince Alfred Hospital (M Daley); Sir Charles Gairdner Hospital (B Roberts); St John of God Hospital, Subiaco (S Webb); St Vincent’s Hospital, Melbourne (J Santamaria); Sydney Children’s Hospital (J Young); The Children’s Hospital at Westmead, Sydney (M Festa); The John Flynn Private Hospital (R Holland); The Prince Charles Hospital (D Mullany); The Queen Elizabeth Hospital (P Williams); The Townsville Hospital (M Corkeron); The Wollongong Hospital (M Gales); Westmead Hospital (A Banerjee); Women’s and Children’s Hospital, Adelaide (M Yung); *Austria:* University Hospital Innsbruck (N Mutz, M Hiesmayr); General Hospital (P Faybik); Hospital Hietzing (R Fitzgerald); Krankenhaus Barmherzige Brüder Linz (F Firlinger); Krankenhaus Der Barmherigen Brueder Wien (G Zasmeta); Krankenhaus Der Barmherzigen Brüder St. Veit (M Zink); Krankenhaus Der Barmherzigen Schwestern Linz (W Sieber); Krankenhaus Steyr (J Hildegard); Landeskrankenhaus Klagenfurt (R Bakondy); Landeskrankenhaus Stolzalpe (J Schlieber); Landeskrankenhaus Deutschlandsberg (G Filzwieser); Medical University Innsbruck (R Beer, M Joannidis); Medical University of Vienna (T Staudinger); Otto-Wagner Hospital (R Schuster); Unfallkrankenhaus Meidling Der Auva (W Scherzer); University Hospital (K Smolle); Wilhelminenspital (S Fitzal); *Bangladesh:* Central Hospital Limited (R Manzoor); *Belgium:* A.I.T. (J Brunain); Ambroise Paré (A Dive); Asz-Aalst (G Huylenbroeck); Az Groeninge Kortrijk (M Van der Schueren); Az Maria Middelares (H 't kindt); Az Sint Jozef Malle (E Slock); Az Sint Lucas (D Rijckaert); Az St Augustinus (J Raemaekers); Az St Jan Av (M Bourgeois); Az Vesalius (I Van Cotthem); Az Damiaan Oostende (G Nackaerts); C.H.N.D.R.F. (D Gusu); Centre Hospitalier de Mouscron (P Gadisseaux); CH Libramont (V Olivier); Chirec - Braine-L'Alleud (H Lignian); CHPLT Verviers (P Michel); CHR Citadelle (V Fraipont); CHR Haute Senne Soignies (M Van der Stappen); CHR St Joseph Mons-Warquignies (F Forêt); CHU Brugmann (D De Bels, J Devriendt, J Massaut); CHU Charleroi (P Biston); CHU Saint-Pierre (A Roman); CHU Sart Tilman, Liège (B Lambermont); Clinique Sainte Elisabeth (A De Meulder); Clinique Notre Dame (V Frederic); Clinique Notre-Dame de Grâce (T Sottiaux); Clinique Saint Luc, Bouge (P Ruyffelaere); Cliniques de L'Europe, St-Michel (V Collin); Cliniques de L'Europe, Ste Elisabeth (S Anane); Hôpital Francais (P Kleiren); Hôpital Saint-Joseph (M Simon); Hornu (S Machayekhi); Imeldaziekenhuis (E Frans); Institut Jules Bordet (G Leroy, T Berghmans); Jan Yperman Hospital (R Joseph); Olv Ter Linden Ziekenhuis, Knokke (J Eerens); Saint Luc University Hospital (PF Laterre); Sint Augustinus, Veurne (B Lagrou); St Vincent (R Rutsaert); St-Jozefkliniek Bornem-Willebroek (W Pisarek); UCL Mont-Godinne (A Dive); Universitair Ziekenhuis Gent (J De Waele); University Hospital Brussels (H Spapen); University Hospital of Liege (P Damas); Erasme University Hospital (JL Vincent); ZNA Stuivenberg (M Malbrain); *Belize:* Universal Health Services, Medical Center (J Hidalgo); *Brazil:* Bandeirantes Hospital (M Baptista); Barra Dor Hospital (D Salgado); Biocor Instituto (M Braga); Casa de Saude Sao Jose Caxias (C Avila); Centro Hospitalar Unimed (G Westphal); Centro Integrado de Atençaõ À Saúde -Unimed Vitória (E Caser); Clínica São Vicente da Gávea (A Alves); Complexo Hospitalar Santa Casa de Porto Alegre (G Friedman); Erasto Gaertner Hospital (M Luz); Federal University of Sao Paulo (M Assuncao); Fundacao Hospital de Clinicas Gaspar Vianna (H Reis); Fundação Hospitalar Do Estado de Minas Gerais - Fhemig (A Gomes); Fundação Pio Xii (U Silva); UNIFESP (W Nogueira Fh); Hopital das Clínicas - FMUSP (S El-Dash); Hospital Padre Albino-Faculdade de Medicina de Catanduva (J Valiatti); Hospital Alberto Cavalcanti (A Barbosa); Hospital Badim (C Coelho); Hospital Cardiotrauma Ipanema (M Knibel); Hospital Carlos Fernando Malzoni (C Minelli); Hospital Da Cidade de Passo Fundo (J Caovilla); Hospital das Clínicas da Faculdade de Medicina de Ribeirão Preto da Universidade de São Paulo (G Teixeira); Hospital das Clínicas, Universisty of São Paulo (A Hovnanian); Hospital das Nacoes (A Rea-Neto); Hospital de Base-Famerp (SL Lobo); Hospital de Clínicas Mario Lioni (M Lugarinho); Hospital de Clínicas Niterói (P Souza); Hospital de Doenças Tropicais de Goiânia (D Ferreira); Hospital do Cancer/Uopeccan (P Duarte); Hospital do Trabalhador (M Oliveira); Hospital dos Servidores do Estado Rio de Janeiro (J Marques); Hospital E Maternidade São José (R Machado); Hospital Estadual Diadema (P Rehder); Hospital Estadual do Grajau-Unisa (S Mataloun); Hospital Evangelico (M Grilo); Hospital Evangelico do Rio de Janeiro (P Quesado); Hospital Geral de Pedreira (M Moock); Hospital Geral de São Mateus (F Ferreira); Hospital Geral Roberto Santos (J Teles); Hospital Israelita Albert Einstein (E Silva); Hospital Israelita Albert Sabin (C Coelho); Hospital Júlia Kubitschek (A Morais); Hospital Mater Dei (F Carvalho); Hospital Memorial Arthur Ramos (M Wanderley); Hospital Meridional (M Velasco); Hospital Moinhos de Vento (N Brandão da Silva); Hospital Municipal São José (J Feijó); Hospital Nossa Senhora Da Salete (P Duarte); Hospital Pasteur (V Souza Dantas); Hospital Português (J Teles); Hospital Pró-Cardíaco (R Costa Filho); Hospital Quinta D'Or (A Japiassu); Hospital Regional Antônio Dias (D Villela); Hospital Regional de Barbacena (C Santos); Hospital Salvador (R Passos); Hospital Samaritano (R Alheira-Rocha); Hospital Santa Izabel (R Silva); Hospital Santa Paula (J Houly); Hospital Sao Cristovao (J Aldrighi); Hospital São Lucas (R Hatum); Hospital São Lucas da PUCRS (F Suparregui Dias); Hospital São Luiz - Unidade Itaim (L Ferreira); Hospital São Rafael (L Ferro); Hospital São Vicente de Paulo (J Gomez); Hospital Universitário Clementindo Fraga Filho - Ufrj (R Fleury); Hospital Universitario da Universidade Federal do Rio de Janeiro (C David); Hospital Universitário de Santa Maria (T Resener); Hospital Universitário do Oeste do Paraná (P Duarte); Hospital Universitário Lauro Wanderley - UTI Adulto (C Mendes); Hospital Universitario Regional de Maringa (A Germano); Hospital Vita Curitiba (M Oliveira); Hospital Vivalle (F De marco); Instituto de Espquisa Clinica Evandro Chagas (A Japiassu); Instituto Do Coração - HC-FMUSP (S Lage); Instituto Nacional de Cancer (J Salluh); Irmandade Santa Casa de Misericordia de Porto Alegre (A Torelly); Luxemburgo Hospital (R Sad); Mternidade Odete Valadares (A Barbosa); Prontocor Lagoa (G Oliveira); Samaritano Hospital (R Lima); Santa Casa Da Misericórdia de São João del Rei (J Paranhos); Santa Casa de Misericordia de Passos (M Oliveira); Santa Casa de Porto Alegre (M Rocha); São Sebastião Hospital (W Bitencourt); Universidade Federal Do Parana (A Rea-Neto); University of Londrina (C Grion); University of Sao Paulo (D Forte); Uti Da Disciplina de Clínica Médica-Unifesp (H Guimarães); Vitória Apart Hospital (C Piras); *Bulgaria:* Mbal Ruse (L Stephanova); Multiprofile Hospital of Active Treatment, Ruse (L Lyubenov); Uh St. Ekaterina (G Tsarianski); Univerisity Hospital (G Dimov); *Canada:* Capital Health-Queen Elizabeth II Health Sciences Centre (R Green); Centre Hospitalier Régional de Lanaudière (J Levasseur); Children’s Hospital of Eastern Ontario (R Ward); CHU Sherbrooke (O Lesur); Hôpital Charles Lemoyne (G Poirier); Mount Sinai Hospital (R Wax); Royal Jubilee Hospital (G Wood); St. Joseph’s Healthcare (D Cook); St. Michael’s Hospital (J Marshall); Toronto General Hospital (M Herridge); Toronto Western Hospital (N Ferguson); Victoria General Hospital (G Wood); *Chile:* Clinica Alemana de Santiago (M Espinoza); Clinica las Condes (S Valdés jimenez); Hospital Clínico de la Pontificia Universidad Católica de Chile (A Bruhn); Hospital del Trabajador (J Micolich); Hospital Dr G. Fricke (S Galvez); Hospital El Pino (I Escamilla Leon); *China:* Beijing Chaoyang Hospital (Q Zhan); Beijing Tongren Hospital (Y Xu); Chinense Pla General Hospital (Y Zhao); Fuxing Hospital, Capital Medical University (L Zhang); Guangdong Provincial People’s Hospital (T Qin); Peking Union Medical College Hospital (B Du); Peking University People’s Hospital (M Li); Ren Ji Hospital,Shanghai Jiao Tong University (X Wang); The Affiliated Hospital of Ningxia Medical College of China (Y Jing); The First Affiliate Hospital of China Medical University (Z Zhang); The First Affiliated Hospital of Dalian Medical University (W Xianyao); The First People’s Hospital of Nantong, Jiangsu (F Li); Zhong-Da Hospital and School of Clinical Medicine, Southeast University (Y Congshan); *Colombia:* Clinica General del Norte (C Rebolledo); Clinica Central del Quindio (D Diaz); Clinica Medellin (R Murillo Arboleda); Clinica Saludcoop (C Rebolledo); Clinica Santa Isabel de Valledupar. (A Arias Antun); Fundación Hospital San Carlos (G Montenegro); Fundacion Valle del Lili (M Granados); Hospital Bocagrande de Cartagena (C Duenas); Hospital Departamental de Villavicencio (N Perez); Hospital El Tunal (G Libreros Duque); Hospital San Jose de Bogota (M Coral); Hospital Santa Clara (G Ortiz); *Costa Rica:* Hospital Calderón Guardia CCSS (D Rodriguez); *Croatia:* Hospital for Infectious Diseases (B Barsic); Sveti Duh General Hospital, School of Medicine, Zagreb (M Cubrilo-Turek); University Hospital Centre (I Gornik); University Hospital Zagreb (M Grljusic); *Cuba:* Hospital Universitario Arnaldo Milian Castro (A Caballero lopez); Hospital Universitario Dr. Gustavo Aldereguía Lima (M Iraola ferrer); *Czech Republic:* Centre of Cardivascular and Transplant Surgery (P Pavlik); Charles University Teaching Hospital, Hradec Kralove (J Manak); Charles University Medical School and Teaching Hospital (J Radej); Faculty General Hospital, Charles University Prague (J Belohlavek); Faculty Hospital Brno (P Sevcik); Faculty Hospital Olomouc (L Blahut); General Teaching Hospital of 1St Faculty and Charles University (D Tyl); Horovice Hospital (J Steinbach); Klaudians Hospital (I Herold); Krajska Nemocnice Liberec (I Zykova); Nemocnice V Usti Nad Orlici (D Prchal); St. Anne’s University Hospital Brno (T Bartosik); University Hospital Brno (M Kolarova); University Hospital Olomouc (R Hájek, J Kohoutová, O Marek); University Hospital Ostrava (P Hon); University Hospital Plzen (I Chytra); *Denmark:* Århus University Hospital (H Betsch); Næstved Hospital (B Fogh); Rigshospitalet (K Espersen); Sygehus Fyn (K Jacobsen); Vejle Sygehus (P Berezowicz); *Ecuador:* Carlos Andrade Marín Hospital (F Guerrero); Clinica La Merced (E Salgado); Hospital Eugenio Espejo (D Barahona); Hospital General de Las Fuerzas Armadas del Ecuador Hg-1 (H Del pozo sanchez); Hospital Metropolitano (M Jibaja); *Egypt:* Dar Alfouad Hospital (A Alansary); *Estonia:* East Tallinn Central Hospital (A Reintam); Tartu University Hospital (J Starkopf); *Finland:* Helsinki University Central Hospital (V Harjola); *France:* AP-HP, CHU Jean Verdier (L Tual); Assistance Publique-Hôpitaux de Marseille, CHU Nord (M Leone); Centre Hospitalier Dunkerque (M Serge); Centre Hospitalier Universitaire (P Michel); Centre Hospitalier (O Leroy); Centre Hospitalier D'Auch (L Mallet); Centre Hospitalier de Blois (B Marc); Centre Hospitalier de Fougères (D Dormoy); Centre Hospitalier de Niort (H Pascal); Centre Hospitalier Dr Schaffner (L Tronchon); Centre Hospitalier du Pays D'Aix (B Garrigues); Centre Hospitalier Region Annecy (C Santré); Centre Hospitalier Universitaire Amiens (H Dupont); Centre Hospitalier Universitaire de Bicêtre (J Duranteau); Centre Hospitalier Universitaire Reims (A Leon); CH Colmar (L Henry); CHG Armentieres (C Canevet); CHU Angers (L Dube); CHU Angers (H Julien); CHU Bicetre (A Nadia); CHU Brest (B Francois); CHU de Bordeaux (J Gérard); CHU Dijon Hopital Général (M Freysz); CHU Hôtel Dieu - APHP (G Remy); CHU Nantes (Y Blanloeil); Clinique Ambroise Paré (P Squara); General Hospital (J Korach); Grenoble University Hospital (M Durand); Groupe Hospitalier du Havre (C Gabriel); Hia Laveran (P Eric); Hopital Antoine Béclère APHP (F Jacobs); Hopital Bichat (R Bronchard); Hôpital Claude Huriez, Centre Hospitalier Régional Universitaire de Lille (E Kipnis); Hopital Cochin Paris (M Moussa); Hôpital de Hautepierre (A Launoy); Hopital de la Croix Rousse (C Guérin); Hôpital Edouard Herriot (P Vanhems); Hôpital Maison Blanche (A Wynckel); Hôpital Raymond Poincaré (B Clair); Hôpital Saint-Louis (E Azoulay); Hôpital Tenon (J Fulgencio); Hôpitaux Civils de Colmar (Y Gottwalles); Hôpitaux Universitaires de Strasbourg (T Krummel); Hospices Civils de Lyon (A Lepape); La Rochelle Hospital (O Lesieur); Lariboisiere University Hospital (D Payen); Poissy Hospital (O Hérvé); Polyclinique Saint André (J Farkas); Rangueil Hospital (P Cougot); Réanimation Chirurgicale (Y Malledant); University Hospital of Bordeaux Haut-Lèvéque (O Joannes-Boyau); *Germany:* Academic Hospital Solingen (T Standl); Ameos Klinikum St.Salvator Halberstadt GMBH (U Sierig); Asklepios Fachkliniken München-Gauting (J Geiseler); Asklepios Klinik Langen (H Hopf); Behandlungszentrum Vogtareuth (M Burgau); Bergmannsheil Bochum (E Conrad-Opel); Bethanien-Krankenhaus (C Hermann); Bundeswehrkrankenhaus Ulm (M Ventzke); Charite/Campus Virchow-Klinikum (T Henneberg); Charite Berlin-Buch (H Loeser); Charité Campus, Mitte (C Spies); Charité Campus, Virchow Klinikum (C Spies); Charite Campus, Virchow (F Esposito); Charité Universitätsmedizin Berlin (H Zuckermann-becker); Clemenshospitl (R Scherer); Dominikus Krankenhaus (A Pauer); Drk-Kliniken Mark Brandenburg (S Kljucar); Drk-Krankenhaus Ratzeburg (K Delfs); Elisabeth-Krankenhaus Essen (E Blank); Ev. Kliniken Bonn Betriebsstätte Waldkrankenhaus (J Busch); Ev.-Freikirchliches Krankenhaus Rüdersdorf (K Wendt); Evang. Krankenhaus Mülheim (J Leßmann); Evangelische Kliniken Bonn Wadkrankenhaus (J Busch); Evangelisches Krankenhaus Bielefeld (F Bach); Friedrich Schiller University, Jena (Y Sakr); Gemeinschaftskrankenhaus Herdecke (T Berlet); Georg-August University of Göttingen (A Kernchen); Georg-August-University of Göttingen (M Quintel); Hanse-Klinikum Wismar (D Holst); Heart clinic of the University of Munich (E Kilger); Helfenstein Klinik (T Holubarsch); Helios Klinik Lengerich (C Raufhake); Helios-Klinikum Berlin-Buch (R, Kuhlen, C Stolt); Helios Klinikum Emil Von Behring (A Lubasch); Helios Klinikum Erfurt Gmbh (A Meier-Hellmann); Helios Klinikum Wuppertal Barmen (G Woebker); Henriettenstift (C Scharnofske); Herz-Jesu-Krankenhaus (M Breyer); Hochtaunus Kliniken Bad Homburg (T Risch); Hospital Links Der Weser (C Manhold); Icu In Drk Kliniken Mark Brandenburg (S Kljucar); J.W. Goethe - University Medical School Frankfurt Am Main (D Meininger); Johanniter Krankenhaus Bonn (C Greive); Johanniter Krankenhaus Stendal Ggmbh (J Rau); Jung-Stilling-Krankenhaus (A Seibel); Katharinenhospital (A Henn-beilharz); Katholisches Krankenhaus Hagen (R Wolbert); Krankenhaus Prignitz Gemmeinnützige GMBH (T Scherke); Klinik Am Eichert (J Martin); Klinik Für Herzchirurgie (M Rudolph); Klinik Füranästhesie, Operative Intensivmedizin U. Schmerztherapie (J Gleißner); Kliniken Ludwigsburg-Bietigheim GMBH (M Wolf); Kliniken Maria Hilf (F Schleibach); Klinikum Augsburg (U Jaschinski); Klinikum Bad Salzungen (A Lunkeit); Klinikum Darmstadt (M Welte); Klinikum Der J.W. Goethe-Universität (T Bingold); Klinikum Der Stadt Wolfsburg (K. Sydow); Klinikum Emden (K Kogelmann); Klinikum Forchheim (F Fischer); Klinikum Fuerth (B Fischer, M Schmid); Klinikum Grosshadern, Universität München (M Klein); Klinikum Harlaching Städtisches Klinikum Muenchen (A Bechtold); Klinikum Hildesheim (K Bodmann); Klinikum Kaufbeuren (J Klasen); Klinikum Landsberg (H Meyrl); Klinikum Lippe - Detmold (J Goetz); Klinikum Ludwigsburg (G Geldner); Klinikum Luedenscheid (T Helmes); Klinikum Meiningen GMBH (N Jensen); Klinikum Minden (H Eickmeyer, W Lengfelder); Klinikum Nürnberg (B Langenstein); Klinikum Rechts Der Isar (R Bogdanski); Klinikum Rechts Der Isar Der Technischen Universität München (S Jelen-Esselborn, A Umgelter); Klinikum Region Hannover (F Dörr); Klinikum Region Hannover Krankenhaus Großburgwedel (K Lüttje); Klinikum Region Hannover, Krankenhaus Oststadt-Heidehaus (D Heinemeyer); Klinikum Starnberg (M Uhl); Klinikum Stuttgart - Olgahospital (P Schirle); Klinikum Suedstadt (H Benad); Klinikum Traunstein (M Glaser); Klinikum Uelzen (W Panzer); Klinikum Worms (E Huettemann); Klinikverbund St. Ansgar, Krankenhaus Bassum (R Stierwaldt); Klinikverbund Süd-West (M Schappacher); Knappschaftskrankenhaus Bochum-Langendreer (E Müller); Krankenhaus Freyung (Rural Hospital) (W Stadlmeyer); Krankenhaus Lübbecke (M Fantini); Krankenhaus Mol GMBH Strausberg (B Dummer); Krankenhaus Nordwest (M Thörner); Krankenhaus Nordwest (V Jost); Krankenhaus Reinbek (T Loerbroks); Kreisklinik Trostberg (T Glück); Kreiskrankenhaus Bergstrasse (R Zimmermann); Kreiskrankenhaus Calw (R Clement); Kreiskrankenhaus Mechernich GMBH (R Hering); Kreiskrankenhaus Nagold (T Klinger); Kreiskrankenhaus Rottweil (J Mehl); Kreiskrankenhaus St. Marienberg Helmstedt (H Polozek); Leopoldina-Krankenhaus (A Rothhammer); Ludmillenstift (R Seidler); Lukas-Krankenhaus Bünde (P Lorenz); Lungenfachklinik Amsee Waren Mueritz (M Lutze); Marienhospital Bruehl (M Euler); Marienkrankenhaus Schwerte (M Heintz); Martin Luther Universität Halle (M Winkler); Medizinische Klinik (M Angstwurm); Mlu Halle-Wittenberg (K Krohe); Mueritz-Klinikum Waren (T Treu); Neurological Intensive Care Unit (T Steiner); Oberschwabenklinik Wangen (S Locher); Orthopädische Klinik Markgröningen (A Walz); Ostalb-Klinikum Aalen (P Zahn); Otto-Von-Guericke Universität Magdeburg (W Brandt); Scivias-Krankenhaus St. Josef (M Marks); Ska-Bileelfeld-Mitte (F Henning); St. Antonius Hospital (U Janssens); St. Hildegardis Krankenhaus Mainz (M Luethgens); St. Johannes Krankenhaus (W Theelen); St. Johannes-Hospital (M Sydow); St. Johannes-Hospital (M Weber); St. Josef-Hospital, Ruhr-Universität Bochum (A Meiser); St. Josefs-Hospital (C Deutschmann); St. Joseph Krankenhaus (C Buttner); St.-Marien Hospital Lünen (M Jokiel); St. Marienhospital Hamm (C Bozzetti); St. Vincentius Kliniken (B Jürgen); St.-Elisabeth Krankenhaus Köln-Hohenlind (F Fiedler); St.-Vincentius-Krankenhaus Speyer (K Wresch); Städtischen Kliniken Neuss - Lukaskrankenhaus (A Kremer); Städtisches Klinikum Karlsruhe (H Bleier); Städtisches Klinikum Wiesbaden, Dr.-Horst-Schmidt-Kliniken Hsk (M Rueckert); Staedtisches Klinikum Guetersloh (H Ditter); Staedtisches Klinikum Muenchen Gmbh - Klinikum Harlaching (C Peckelsen); Staedtisches Klinikum Muenchen Gmbh/Klinikum Bogenhausen (P Friederich); Staedtisches Klinikum München - Klinikum Neuperlach (K Weber); Tübingen University Hospital (W Krueger); Ubbo-Emmius-Klinik Aurich (R Lowack); Überlingen Hospital (A Michalsen); Uniklinikum Dresden (M Ragaller); Universitaetsklinikum des Saarlandes (A Groeschel); Universitaetsklinikum Mannheim (T Friedrich, U Hoffmann); Universität Rostock (M Hinz); Universitätsklinikum Der Martin-Luther-Universität Halle-Wittenberg (A Christel); Universitätsklinikum Dresden Carl Gustav Carus (M Ragaller); Universitätsklinikum Leipzig Aör (T Hartwig); Universitätsmedizin Berlin Charité Campus Benjamin Franklin (S Vögeler); University (M Weiss); University Childrens Hospital Hauner (K Reiter); University Hospital (T Schwab); University Hospital Cologne (U Trieschmann); University Hospital Dusseldorf (D Kindgen-milles); University Hospital Giessen and Marburg Gmbh - Giessen (J Engel); University Hospital Lübeck (B Sedemund-adib); University Hospital Mainz (M Lauterbach); University Hospital Marburg (M Max); University Hospital Muenster (T Volkert); University Hospital of Essen (C Waydhas); University Hospital of Mannheim (S Hien); University Hospital of Munich, LMU (J Briegel); University Hospital of Regensburg (V Guralnik); University Hospital Rwth Aachen (N Zoremba); University Hospital Tübingen (R Riessen); University Hospital Würzburg (W Müllges); University Medical Center Hamburg-Eppendorf (A Nierhaus); University of Erlangen (R Strauss); University of Freiburg (S Utzolino); University of Giessen (J Thul); University of Greifswald (P Abel, M Gründling, W Keßler); University of Heidelberg (K Scheuren); University of Leipzig (E Lothar, U Kaisers, D Schmitt, D Schneider); University of Rostock (D Vagts); University of Saarland (H Rensing); University Hospital Essen (B Schoch); Universty Hospital (K Kopp); Vivantes - Klinikum Neukoelln (H Gerlach); Vivantes Klinikum Prenzlauer Berg (M Corea); Vivantes-Klinikum Am Urban (A Uhrig); Westkuestenklinikum Heide (S Schroeder); Westküstenklinikum Heide (F Jordan); Westpfalz-Klinikum Kaiserslautern (T Huber); Zentralöklinikum Augsburg (M Bittinger) *Greece:* Ahepa University Hospital (E Sofianos); Athens University Medical School (A Armaganidis); Evangelismos Hospital (C Routsi); G.Papanikolaou (M Bitzani); General Hospital of Rethymno (A Chalkiadaki); Henry Dunant Hospital (A Michalopoulos); Hippokrateion Hospital Thessaloniki (E Mouloudi); Kat General Hospital (E Ioannidou); Kat Hospital (P Myrianthefs); Kat Hospital, Athens (D Koulenti); Konstantopoulio General Hospital (I Karampela); Lamia General Hospital (G Kyriazopoulos); Red Cross Hospital of Athens (K Mandragos); Thriassio Hospital of Eleusis (P Clouva-molyvdas); University Hospital of Ioannina (A Moraiti); University Hospital of Alexandroupolis (I Pneumatikos); University Hospital of Rion, Patras (K Filos); University Hospital of Thessaly (Larissa) (E Zakynthinos); University of Athens, Medical Shcool (A Kotanidou); Xanthi General Hospital (A Vakalos) *Hong Kong:* Northern District Hospital (A Cheng); Princess Margaret Hospital and Yan Chai Hospital (T Buckley); The Chinese University of Hong Kong (C Gomersall) *Hungary:* National Institute of Neurosurgery (K Kiss); Péterfy Hospital Budapest (P Tamási); Saint George Hospital Hungary (A Sarkany); Semmelweis University (A Csomos); University of Szeged (É Zöllei) *India:* Advanced Medicare Research Institute (S Todi); B.D.Petit Parsee General Hospital (F Udwadia); Bhailal Amin General Hospital (R Shah); Bombay Hospital (P Amin); Breach Candy Hospital Trust (F Udwadia); Care Hospitals (S Samavedam); Christian Medical College (A Mathai); Cumballa Hill Hospital & Heart Institute (M Patil); Deenanath Mangeshkar Hospital (S Jog); Dr. S. N. Medical College (M Gurjar); Escorts Heart Institute & Research Centre (M Vats); Fortis Healthcare (A Varma); Global Hospitals (P Gopal); Hinduja Hospital & Medical Research Center (F Kapadia); Indraprastha Apoolo Hospitals (R Chawla); Jehangir Hospital (S Iyer); Kalinga Hospital (S Sahu); Kasturba Hospital (C Bakshi); Lokmanya Care Hospital (D Ambike); Max Super Speciality Hospital (D Govil); Medical Trust Hospital, Cochin (V Karipparambath); Nh Hospital (J Chacko); Ruby Hall Clinic (P Sathe); Rungta Hospital (N Rungta); Saifee Hospital (C Jani); Seth Ramdas Shah Memorial Hospital & Research Centre (A Bhome); Shree Medical Foundation (S Prayag); Sir Ganga Ram Hospital (S Ray); Sundaram Medical Foundation (R Rajagopalan); Tata Memorial Hospital (J Divatia); Wockhardt Hospital (R Da costa); Wockhrdt Hospital (T Shyam Sunder) *Indonesia:* Bintaro International Hospital (P Wibowo); Hasan Sadikin Hospital (T Maskoen); Pantai Indah Kapuk Hospital (T Sugiman) *Islamic Republic of Iran*: Imamreza Hospital (S Nowruzinia); Laleh Hospital (A Lotfi); Tehran University of Medical Sciences (A Mahmoodpoor) *Ireland:* Amnch (M Donnelly); Cork University Hospital (D Breen); Mater Misercordiae University Hospital (S Ng); University Hospital Galway (J Bates) *Israel:* Hadassah Medical Center (C Sprung); Haemek Medical Center (A Lev); Kaplan Medical Center (E Kishinevsky); Rabin Medical Center (J Cohen); Soroka Medical Center (S Sofer) *Italy:* A.O Niguarda (S Vesconi); A.O. Ospedale Di Circolo Di Busto Arsizio (S Greco); A.O. Treviglio-Caravaggio (M Borelli); Anestesia E Rianimazione 2 Prof.De Gaudio (P Cecilia); Arnas Ospedale Civico (M Sapuppo); ASL 10 (A Lazzero); ASL 10 Florence Hospital San Giovanni Di Dio (V Mangani); Azienda Ospedaliera Desenzano (N Petrucci); Azienda Ospedaliera Di Melegnano (M Minerva); Azienda Ospedaliera G. Rummo (E De blasio); Azienda Ospedaliera Polo Universitario San Paolo (S Marzorati); Azienda Ospedaliera Santa Maria Alle Scotte (R Rosi); Azienda Ospedaliera Universitaria P.Giaccone Policlinico (A Giarratano); Azienda Ospedaliera-Universitaria Udine (O Margarit); Azienda Ospedaliero -Universitaria (A Guberti); Azienda Ospedaliero-Universitaria S.M.Misericordia (S Scolz); Clinica San Gaudenzio (E Stelian); Fondazione IRCCS Policlinico San Matteo (V Emmi); Fondazione Ospedale Maggiore Policlinico, Mangiagalli Regina Elena (M Caspani); Fondazione Poliambulanza (A Rosano); H San Gerardo (C Abbruzzese); Hospital Panico Tricase (S Colonna); Humanitas Gavazzeni (R Ceriani); II Faculty of Medicne I University of Rome- Osp. S.Andrea (R De Blasi); S. Salvatore Hospital (L Panella); IRCCS Casa Sollievo Della Sofferenza (F Borrelli); Istituto Nazionale Tumori Regina Elena (P Lorella); KH Brixen (H Ruatti); Osepdali Riuniti Di Ancona (C Munch); Ospedale "Ca Foncello" - Treviso (Italia) (C Sorbara); Ospedale "Santa Croce" - ASL 8 (G Fiore); Ospedale Bufalini-Cesena (A Chieregato); Ospedale Di Circolo E Fondazione Macchi (V Conti); Ospedale Di Massa (A Guadagnucci); Azienda USL Piacenza (M Pizzamiglio); Ospedale Ferrarotto (M Locicero); Ospedale Maggiore Ausl Bologna (I Marri); Ospedale Maggiore Policlinico Milano (A Sicignano); Ospedale Maggiore Policlinico, Mangiagalli E Regina Elena, IRCCS Milano (V Conte); Ospedale Mugello Azienda Sanitaria Firenze (R Oggioni); Ospedale Niguarda Ca Granda, Milano (A De Gasperi); IRCCS Centro di Riferimento Oncologico della Basilicata (P De Negri); Ospedale Provinciale Pistoia (G Santagostino); Ospedale S. Gerardo (R Fumagalli); Ospedale San Raffaele (G Marino); Ospedele Vittorio Emanuele (G Castiglione); P.O. San Severo Asl Fg (D Sforza); S. Camillo Hospitql (N Giuseppe); San Martino Hospital (M Bassetti); Seconda Università Degli Studi Di Napoli (F Ferraro); Sesto San Giovanni Hospital (S Clementi); Teaching Hospital Careggi (D Alessandro); Terapia Intensiva - Aso S. Giovanni Battista Di Torino - Ospedale Molinette (P Cotogni, MV Ranieri); Università Cattolica (M Antonelli); Universita' Cattolica Del S. Cuore (L Martinelli); University-Hospital Careggi, Florence, (L Gianesello); University Hospital Policlinico Di Catania (A Gullo); University of Rome "La Sapienza" (A Morelli); UTI Trapianti (G Biancofiore); University of Udine (G Della Rocca) *Japan:* Kyoto Prefectural University of Medicine (S Hashimoto); Nagoya University Hospital (M Onodera); Oosaka-Fu Saiseikai Suita Hospital (A Kobayashi); Sanai Hospital (T Shinozuka); Tokushima University School of Medicine (H Imanaka); Tokyo Medical University, Hachioji Medical Center (T Ikeda); Tokyo Women’s Medical University (A Yaguchi) *Latvia:* Hospital of Traumatology and Orthopedics (I Misane); 7th Hospital of Riga (A Piebalga) *Lebanon:* Lebanese Canadian Hospital (A Moughaghab) *Lithuania:* Medicine University of Kaunas (V Pilvinis); Vilnius University Emergency Hospital (S Vosylius); Vilnius University Hospital Santariskiu Clinics (M Balciunas, G Kekstas) *Luxembourg:* Centre Hospitalier de Luxembourg (H Margaret); Clinique Ste Thérèse (M Klop) *Macedonia*: Clinic For Infectious Diseases (K Grozdanovski); General Hospital Stip (B Eftimova) *Malaysia:* Faculty of Medicine, Universiti Kebangsaan Malaysia (S Wafa); Hospital Pulau Pinang (C Lim); Hospital Tengku Ampuan Afzan, Kuantan, Pahang (M Mat nor); Kuala Lumpur Hospital (L Tai); National Heart Institute (S Syed Mohd Tahir); Sarawak General Hospital (N Idris); Sultanah Aminah Hospital (C Tan) *Malta:* St Luke’s Hospital (M Borg); *Mexico:* Angeles Metropolitano Hospital (E Manzo); Centro Medico Lic.Adolfo Lopez Mateos (H Gutierrez Morales); Hgr 25 Imss Zaragoza (P Miguel); Hospital Angeles Clinica Londres (A Villagomez); Hospital Angeles del Carmen (A Bassols); Hospital Civil de Guadalajara "Fray Antonio Alcalde" (G Aguirre); Hospital Español de México (U Cerón); Hospital General Bernardo J. Gastelum (J Lopez ramos); Hospital General del Estado "Dr Ernesto Ramos Bours", Hermosillo Sonora Mexico (J Monjardín); Hospital General Regional de Leon (E Bermudez Aceves); Hospital General Reynosa (F Gonzalez Salazar); Hospital Juan I.Menchaca Hospital Civil de Guadalajara (D Rodriguez Gonzalez); Hospital Juárez de México (M Poblano-Morales); Hospital Mèdica Sur (F Ramirez); Hospital O´horan (M Cetina); Hospital Privado de Hermosillo (J Navarro); Hospital Regional 1° Octubre, Issste (A Villagomez Ortiz); Hospital San Jose Tec Monterrey (V Sanchez); Hospital Universitario "Dr. Jose E. Gonzalez" (U Chavarria); IMSS (O Fernandez-Ponce); Iner (H Serna secundino); Instituto de Salud del Estado de Aguascalientes (O Leonardo); Instituto Mexicano del Seguro Social (R Diego Manuel, J Mijangos); Issemym Medical Center (G Vazquez de Anda); Mexican Red Cross (E Martin); Ocq Hospital (P Gutierrez); Secretaria de Salud del Gobierno del Distrito Federal (I López Islas); Servicios de Salud En Yucatan (L Soberanes) *Montenegro*: Clinical Center of Montenegro (P Ljubica) *Morocco:* Chu Ibn Sina (A Sbihi); Polyclinique CNSS Derb Ghallef (B Ouahid); Réanimation Médicale, Hôpital Ibn Sina (M Naoufel) *Netherlands:* Academic Medical Center (A De Pont); Amphia Hospital (P Rosseel); Antoni Van Leeuwenhoek Ziekenhuis (J Ten Cate); Beatrix Zienhuis Rivas Zorggroep (G Van Berkel); Canisius Wilhelmina Ziekenhuis (S Corsten); Erasmus Mc University Medical Center (J Bakker); Hagaziekenhuis (J Vogelaar); Hofpoort Hospital Woerden (H Blom); Isala Clinics (H Kieft); Medical Center Leeuwarden (M Kuiper); Medisch Spectrum Twente (A Gille); Radboud University Nijmegen Medical Centre (P Pickkers); Rode Kruis Ziekenhuis (J Vet); Slingeland Ziekenhuis (J Ammann); Spaarneziekenhuis (S Den Boer); St. Antonius Ziekenhuis (R Wesselink); St.Elisabeth Hospital (B Speelberg); Twenteborg Hospital Almelo (C Pham); University Medical Center, Groningen (M Rodgers); University Hospital Maastricht (D Bergmans); Vu University Medical Center (J Groeneveld) *New Zealand:* Auckland City Hospital (C McArthur); Auckland City Hospital (R Parke); Christchurch (J Mehrtens); Dunedin Hospital (L Celi); Hawke’s Bay Hospital (R Freebairn); Middlemore Hospital (N Rankin); Nelson Marlborough District Health Board (C Heffernan); Palmerston North Hospital (G McHugh); Starship Children’s Hospital (J Beca); Waikato Hospital (F Van Haren); Wellington Public Hospital (B Barry); Whangarei Base Hospital (M Kalkoff) *Norway:* Aker University Hospital (R Loevstad); St Olavs University Hospital (P Klepstad); Sykehuset Asker Og Bærum Hf (P Erno); Sykehuset I Vestfold Hf , Toensberg (A Junker) *Pakistan:* Armed Forces Institute of Cardiology (S Naqvi); Jinnah Hospital Lahore Pakistan (I Javed) *Panama*: Complejo Hospitalario Metropolitano (J Sinclair) *Peru:* Hipolito Unanue Hospital (R Rivera); Hospital Regional Honorio Delgado (C Chavez); Hospital Alberto Sabogal Sologuren (Z Donayre Taber); Hospital Dos De Mayo (R Quispe Sierra); Hospital Edgardo Rebagliati Martins (J Muñoz); Hospital Maria Auxiliadora (J Galvez Ruiz); Hospital Nacional Almanzor Aguinaga Asenjo Essalud Chiclayo (J Fang Li); Hospital Nacional Arzobispo Loayza (M Candiotti Herrera); Hospital Víctor Lazarte Echegaray (A Arroyo); Instituto de Salud del Niño (R Becerra); Navy Hospital (J Meza); Peruvian Air Force Central Hospital (M Mayorga) *Poland:* 4th Military Clinical Hospital (P Garba); Academic Centre for Maritime and Tropical Medicine AMG (J Kot); Barlicki University Hospital, University of Medical Science, Lodz, (T Gaszynski); Boleslaw Szarecki Teaching Hospital No. 5 of The Medical University In Lodz (M Piechota); Clinical Hospital No 2 (S Renata); Collegium Medicum Jagiellonian University (P Müller); Institute of Cardiology (J Stepinska); J. Brudzinski’s Hospital In Gdynia (K Jacek); Jagiellonian University (T Cieniawa); Karol Marcinkowski University of Medical Sciences (A Mikstacki, B Tamowicz); Poznan University of Medical Sciences (A Bartkowska-Sniatkowska); Silesian University of Medicine (E Karpel); University Hospital Bydgoszcz Cm Umk (K Kusza); University Hospital No 2 (P Smuszkiewicz); University Hospital Warsaw (M Mikaszewska-Sokolewicz); Wojewodzki Szpital Specjalistyczny (R Goraj); Wroclaw Medical University (A Kubler) *Portugal:* Centro Hospitalar Alto Ave (A Bártolo); Centro Hospitalar Cova Da Beira (M Castelo-Branco Sousa); Centro Hospitalar Trás os Montes e Alto Douro (F Esteves); CHLO-Hospital S Francisco Xavier (A Martins); H S João (T Oliveira); Hospital CUF Infante Santo (P Ponce); Hospital Curry Cabral (L Mourão); Hospital da Luz (C Febra); Hospital de Egas Moniz (E Carmo); Hospital de S. José (V Lopes); Hospital de São Francisco Xavier (P Póvoa); Hospital de São José (A Rezende); Hospital Divino Espirito Santo (H Costa); Hospital do Litoral Alentejano (P Moreira); Hospital Dr. José Maria Grande, Portalegre (F Pádua); Hospital Fernando Fonseca (A Leite); Hospital Garcia de Orta (E Almeida); Hospital Geral de Santo António (M Alves); Hospital de Pulido Valente (A Sousa, L Telo); Hospital de S. João (C Dias, J Paiva); Hospital de São Bernardo (R Ribeiro); Hospital de São Sebastião, EPE (P Amaro); Hospital Geral de Sto Antánio (A Carneiro); Hospital de St. António dos Capuchos (R Moreno, Ricardo Matos, Susana Afonso); Instituto Português de Oncologia de Lisboa (M Bouw); Hospital de St Maria (C França) *Qatar:* Alkhor Hospital (A Ibrahim); Romania: "Maria Sklodowska Curie" Children’s Emergency Hospital, Bucharest (R Tabacaru); Department Public Hospital (V Ionita); Fundeni Institute (D Tulbure); Institute of Cardiovascular Disease (D Filipescu); Institutul de Boli Cardiovasculare Si Transplant Tg. Mures (S Pascanu); Spitalul "Sf. Spiridon" (I Grigoras); University Emergency County Hospital (S Copotoiu) *Russia:* Bakoulev Scientific Center For Cardiovascular Surgery (D Popov); City Clinical Hospital (E Lebedev); City Hospital (I Olga); City Hospital #7 (A Yaroshetskiy); Clinical Hospital N°40 (T Lugovkina); Ekaterinburg. University Hospital N°40 (B Dmitry); Izhevsk State Medical Academy (O Malinin); Moscow Children’s Surgery Institut (A Lekmanov) *Saudi Arabia:* Alawi Tunsi Hospital (M Abulmagd); King Abdulaziz Medical City (Y Arabi); King Abdulaziz University Hospital (J Alhashemi); King Fahad Specialist Hospital Dammam (A Ali); King Faisal Specialist Hospital (K Maghrabi); Kingdom Hospital (A Debek); Riyadh Millitary Hospital (M Malik) *Serbia:* Clinical Center Nis (R Jankovic); Clinical Centre of Serbia (I Palibrk); Dedinje Cardiovascular Institute (V Maravic-stojkovic); KBC Bezanijka Kosa (V Malenkovic); Military Medical Academy, Belgrade (M Surbatovic); Unuiversity of Belgrade (V Bumbasirevic) *Singapore:* Changi General Hospital (N Lim); Kk Hospital (T Loh); Tan Tock Seng Hospital (H Tan) *Slovakia:* Fakultna Nemocnica Nitra (H Sekeresova); FNSP Bratislava-Hospital Ruzinov (J Koutun); Madical Faculty Hospital Kosice (J Firment); Nusch Bratislava (P Malik); Reiman Hospital (S Trenkler) *Slovenia:* Clinic Center Ljubljana (I Muzlovic); General Hospital Novo Mesto (L Kosec, B Ozek); General Hospital Slovenj Gradec (D Kasnik); University Clinic of Respiratory and Allergic Diseases (V Tomic); University Clinical Center Ljubljana (R Knafelj); University Medical Center Ljubljana (V Svigelj) *South Africa:* 1 Military Hospital (H Du Plessis); Groote Schuur Hospital (R Raine); Johannesburg Hospital (S Bhagwanjee, G Richards, J. Scribante); Johannesburg Hospital Trauma Unit (J Goosen); Unitas Hospital (J De Jager); Wits Donald Gordon Medical Center (G Schleicher) *Spain:* Althaia (O Rubio); Bellvitge University Hospital (R Mañez); Centro Medico Delfos (M Burgueño Campiñez); Clinica Moncloa (M Alvarez); Clinica Rotger (R Jorda); Clinica Santa Elena (E Naveira-Abeigón); Clinica Universitaria de Navarra (P Monedero); Complejo Hospitalario de Pontevedra (E Alemparte-Pardavila); Fundacion Hospital Alcorcon (S Garcia del Valle); Fundacion Jimenez Diaz (C Perez Calvo); H Vall Hebron (M Palomar); H.U. Virgen de Las Nieves- H. Traumatología Y Rehabilitación (F Guerrero); Hospital "Virgen de La Concha" - Zamora (A Caballero Zirena); Hospital Arnau de Vilanova (M Arribas); Hospital Can Misses (E Bustamante Munguira); Hospital Casa de Salud (J Ruiz); Hospital Central de Asturias (L Iglesias); Hospital Clinic (E Zavala); Hospital Clinic de Barcelona (M Valencia); Hospital Clinico San Carlos (A Blesa Malpica); Hospital Clinico San Carlos (F Martinez-Sagasti, M Nieto); Hospital Clinico Universitario (G Aguilar); Hospital Clinico Universitario de Santiago (F Martinon-Torres); Hospital Comarcal Vinaros (C Lorente); Hospital de Navarra (J Insausti); Hospital de Antequera (R Vegas Pinto); Hospital de Basurto (I Santos); Hospital de Fuenlabrada (A Escriba); Hospital de Galdakao (P Olaechea); Hospital de La Merced (E Muñoz); Hospital de Manacor (E Antón Caraballo); Hospital de Mostoles (P Galdos-Anuncibay); Hospital de Sagunto (V Lopez Camps); Hospital de Tortosa Verge de La Cinta (F Esteban-Reboll); Hospital del Sas de Jerez (A Estella); Hospital Donostia (L Bocero); Hospital Dr Peset (A Ibañez); Hospital G. Yagüe (L Pueyo); Hospital General (L María Jesús); Hospital General de Asturias (L Iglesias); Hospital General de Ciudad Real (J Silva); Hospital General de Granollers (P Garro); Hospital General de La Palma (L Ramos-gómez); Hospital General de L'Hospitalet (A Rovira); Hospital General de Vic (M Martin Delgado); Hospital General Salud "Obispo Polanco" (J Monton Dito); Hospital General Universitario de Albacete (F Garcia); Hospital General Universitario de Alicante (J Navarro); Hospital General Universitario de Elche (J Latour-Perez); Hospital General Universitario de Guadalajara (A Albaya); Hospital General Universitario Gregorio Marañon (A Bustinza); Hospital Gran Canaria "Dr Negrín" (J Sole-violán); Hospital Marques de Valdecilla (P Ugarte Peña); Hospital Maz (I Yuste); Hospital Parque San Antonio (J De Rojas Román); Hospital Sabadell (J Vallés); Hospital Sant Joan de Déu (E Esteban); Hospital Sant Pau (E Quintana Tort-Martorell); Hospital Santa María del Rosell (M Moreno); Hospital Santa Maria Madre-Complejo Hospitalario de Ourense (V López Ciudad); Hospital Santiago Apostol (A Manzano Ramirez); Hospital Sevilla-Aljarafe (J Sánchez-Olmedo); Hospital Son Llàtzer (M Borges); Hospital Terrassa (J Amador Amerigo); Hospital Torrecardenas (F Guerrero Gomez); Hospital Universitaio 12 de Octubre (J Montejo González); Hospital Universitari de Girona Doctor Josep Trueta (J Sirvent); Hospital Universitari Germans Trias I Pujol (E Mesalles Sanjuan); Hospital Universitario Arnau de Vilanova (F Barcenilla-Gaite); Hospital Universitario de Canarias (N Serrano); Hospital Universitario de Getafe (E Cerdá); Hospital Universitario de Valme (A Lesmes Serrano); Hospital Universitario Doce de Octubre (C Garcia-Fuentes); Hospital Universitario Infanta Crsitina (J Macias Pingarrón); Hospital Universitario Nªsra de Candelaria (E Espinosa); Hospital Universitario Principe de Asturias (M Sanchez Garcia); Hospital Universitario Reina Sofía, Murcia (F Felices); Hospital Universitario Virgen de La Victoria (M de la Torre-Prados); Hospital Univesitario Puerto Real (H Maria Jesus); Hospital Valle del Nalon (V Luis); Hospital Virgen Arrixaca (R Jara); Hospital Xanit Internacional (M Briones Lopez); Hospital Xeral Cies (P Posada); Hu La Paz (B Galvan); Hu La Paz (F Mariscal); Joan Xxiii University Hospital (J Rello); Morales Meseguer (B Gil); Puerta del Mar Universitary Hospital (R Sierra); Rio Hortega University Hospital (J Rico-Feijoo); San Pedro de Alcantara Hospital (C Corcobado Márquez); Servicio Navarro de Salud. Hospital Virgen del Camino (J Izura); Uci H. U. Salamanca (J González); Universitary Hospital Dr. Peset (J Soto Ibáñez) *Sudan:* Soba University Hospital (H Agabani) *Sweden:* Anestesikliniken (P Petersen); Centralsjukhuset Karlstad (L Johansson); University Hospital, Linköping (H Blomqvist, B Peterzén, N Wyon); Göteborg (I Lindström); Kärnsjukhuset Skövde (A Paulsson); Karolinska University Hospital Huddinge (C Agvald-Ohman); Karolinska University Hospital, Solna (J Petersson); Lund University Hospital (H Friberg); Malmoe University Hospital (V Einar); Op/Iva Kliniken (F Hammarskjöld); Ostersund Hospital (M Schindele); Ostra Hospital, Göteborg (S Arvidsson); Sahlgrenska University Hospital (J Sellgren); Söder Hospital (Södersjukhuset) (J Hulting); Sodersjukhuset (J Häggqvist); Sollefteå Hospital (J Rudenstam); Sunderby Hospital (D Lind); The Queen Silvia Children’s Hospital (E Kokinsky); Thoracic Intensive Care, Karolinska Hospital (A Owall); Umeå University Hospital (S Jacobson); University Hospital (H Stiernstrom); University Hospital of Örebro (A Nydahl) *Switzerland:* CHUV, Lausanne (P Eggimann); University of Geneva (R Stocker); Hirslanden Klinik Beau-Site (G Loderer); Kantonsspital Liestal (R Loetscher); Kantonsspital Luzern (A Mehlig); Lindenhofspital (K Heer); Neuchâtel (H Zender); Ospedale Civico Di Lugano (S Cottini); Regional Hospital Mendrisio (A Pagnamenta); Stadtspital Triemli (G Eich); Swiss Paraplegic Centre (P Felleiter); University Hospital Zurich (M Maggiorini); University Hospitals of Geneva (J Pugin) *Taiwan:* Changhua Christian Hospital (W Shu-Hui); Kaohsiung Veterans General Hospital (K Hsieh) *Thailand:* Faculty of Medicine Siriraj Hospital (P Toomtong); Prince of Songkla University (B Khwannimit); The Bnh Hospital (P Kietdumrongwong) *Tunisia:* Children’s Hospital of Tunis (A Khaldi); Hopital Aziza Othmana (A Messadi); Military Hospital of Tunis (I Labbene); Mongi Slim Hospital (N Frikha) *Turkey:* Acibadem Kadikoy Hospital (K Atalan); Acibadem Bakirkoy Hospital (C Ates); Acibadem Bursa Hospital (A Kahveci); Acibadem Kozyatagi Hospital (H Fistikci); Ankara Univercity (A Kaya); Ankara University Medical Faculty, Ibni Sina Hospital (E Ozgencil); Ataturk University Medical Faculty (M Kizilkaya); Dicle University Medical School (M Bosnak); Dokuz Eylul University (H Bodur); Dokuz Eylul University School of Medicine (M Akan); Erciyes University Medical Faculty (M Guven); Gazi University School of Medicine (M Turkoglu); Hacettepe University Hospital (A Topeli); Inonu University Medical Faculty (T Togal); Istanbul Faculty of Medicine (N Uzel); Istanbul Medical Faculty (I Akinci, N Cakar, S Tugrul); Istanbul University Cerrahpasa Medical School (O Demirkiran); Izmir Ataturk Training and Resarch Hospital (T Adanir); Memorial Hospital (K Dogruer); Okmeydani Teaching & Research Hospital (A Turkmen); Okmeydani Teaching & Research Hospital (H Guven); Ondokuz Mayis University, Medical Faculty (F Ulger); Selcuk University Meram Faculty of Medicine (S Kocak) *Ukraine:* Lugansk City Hospital No. 7 (Y Nalapko); Lugansk District Hospital (Y Nalapko) *United Arab Emirates*: Al-Mafraq Hospital (S Rady); Department of Health & Medical Services (A Alsabbah); Dohms (N Elahi); Dubai Hospital (H Al rahma); Tawam Hospital (M Rahman, S Kashef) *United Kingdom:* Aberdeen Royal Infirmary (B Cuthbertson); Addenbrookes Hospital (K Gunning); Barnsley Hospital (Y Myint); Bristol Royal Infirmary (J Bewley); Cambridge University Teaching Hospitals (R Burnstein); Christie Hospital (P Haji-Michael); Dumfries and Galloway Royal Infirmary (D Wrathall); Kent and Canterbury Hospital (L Folan); Freeman Hospital (I Nesbitt); Friarage Hospital Northallerton (A Ratnaparkhi); Frimley Park Hospital (S Pambakian); Glasgow Royal Infirmary (M Booth); Great Western Hospital Swindon (M Watters); Guys and St Thomas' NHS Foundation Trust (T Sherry); Huddersfield Royal Infirmary (U Buehner); Hurstwood Park Neurosurgical Centre (C Barrera Groba); James Paget University Hospital (P Bothma); John Radcliffe Hospital (N George, J Millo; Kettering General Hospital (L Hollos); Lothian University Hospitals Trust (S Mclellan); Macclesfield District General Hospital (J Hunter); Manchester Royal Infirmary (M Garrioch, N O'Keeffe); Medway NHS Trust (N Divekar); Morriston Hospital (S Eggert); New Cross Hospital,Wolverhampton. (S Smith); Newcastle General Hospital (A Vincent); Newham University Hospital Trust (P Withington); NHS Tayside (C Macmillan); Northampton General Hospital (R Webster); Papworth Hospital (A Vuylsteke); Peterborough Hospitals (B Appadu); Princess Royal Hospital (C Barrera groba); Queen Alexandra Hospital, Portsmouth (P McQuillan); Queen Elizabeth Hospital (M Blunt); Queen Elizabeth Medical Center, Birmingham (N Parekh); Rotherham DGH (D William); Royal Berkshire Hospital (C Jones); Royal Blackburn Hospital (A Krige); Royal Bournemouth NHS Foundation Trust (M Schuster-Bruce); Royal Cornwall Hospital (J Boyden); Royal Devon & Exeter NHS Foundation Trust (C Boulanger); Royal Infirmary of Edinburgh (D Swann); Royal Liverpool University Hospital (J Walker); Royal Marsden Hospital (T Wigmore); Royal Shrewsbury Hospital (R Law); Royal Sussex County Hospital (F Baldwin); South Tyneside Hospital (C Muench); Southmead Hospital (S Robinson); St George’s Hospital (A Crerar-Gilbert, A Rhodes); St Helens and Knowsley Hospital (T Mahambrey); St John’s Hospital (L Cameron); St. James’s University Hospital (J Thornton); St. Mary’s Hospital (M Stotz); St. Peters Hospital (M Russell); Stirling Royal Infirmary (A Longmate); Tameside General Hospital (R Kitson); Taunton & Somerset NHS Trust (B Browne); The Hillingdon Hospital (A Thorniley); The James Cook University Hospital (I Gonzalez); Torbay Hospital (M Swart); University College Hospital (M Singer); University Hospital Birmingham (N Gautam); University Hospital of South Manchester (V Prasad); University Hospitals Coventry & Warwickshire Nhs Trust (D Watson); West Middlesex University Hospital (T Szakmany); West Suffolk Hospital (J Cardy); Western Infirmary Glasgow (A Binning); Wexham Park Hospital (R Loveland); Wirral Hospitals Nhs Trust (J Gannon); Wolverhampton Hospital (G Martinelli); Wythenshawe Hospital (P Nightingale); Yeovil District Hospital (J Howes) *United States:* Baystate Medical Center (J Steingrub); Denver Health Medical Center (L Ammons); Emory University Hospital - MICU (M Fisher); Englewood Hospital Medical Center (N Gandhi); Grady Memorial Hospital-Emory University (G Martin); Hospital of The University of Pennsylvania (C Deutschman); LDS Hospital, Salt Lake City (N Dean); Inova Fairfax Hospital (C Michetti); Los Angeles County + University of Southern California Medical Center (H Belzberg); LSUHSC Shreveport (K Hutchinson); Maine Medical Center (T Van der kloot); Mayo Clinic (B Afessa); Mount Sinai School of Medicine (D Kaufman); Nassau University Medical Center (J Iqbal); New York University (D Ost); Northwestern University (M West, R Wunderink); Northwestern University Feinberg School of Medicine Critical Care (S Afifi); Olive View Ucla Medical Center (S Stein); Oregon Health & Science University (D Hagg); Orlando Regional Medical Center (E Jimenez); Penn State Hershey Medical Center (S Blosser); Rochester General Hospital (S Chhangani); Rush University Medical Center (R Kleinpell); Rutland Regional Medical Center (H Reich); Scott and White Hospital (E Fileds); Sharp Memorial Hospital (D Willms); South Texas Veterans Health Care System (P Castellanos-Mateus); St. Joseph Medical Center Fhs (L Melnik); Texas Tech University Health Sciences Center (L Oud); Cardiovascular ICU, The Methodist Hospital, Houston (E Chi, Y Brown); Medical ICU, The Methodist Hospital, Houston (R Halfon); The University of Mississippi Medical Center (A Badr); The University of Texas Health Science Center at San Antonio (M Restrepo, P Castellanos-Mateus); University of Chicago (A Pohlman); University of Cincinnati (R Branson); University of Kansas Hospital (S Simpson); University of Miami School of Medicine (D Kett); University of Michigan (T Jacobs, P Park, W Wahl); University of Texas Health Science Center at San Antonio (C Patricia); University of Toledo Medical Center (J Hammersley, T Papadimos); University of Virginia (R Sawyer); Uthsc At Memphis - Regional Medical Center (Site) (A Freire); Veterans Caribbean Healthcare System (W Rodriguez); Washington Hospital Center (A Ryan); West Suburban Medical Center (B Margolis); Winthrop University Hospital (M Groth) *Uruguay:* Amecom (H Escanda); Comta (J Baraibar); Hopital Policial (D Paciel); Hospital Maciel (H Bagnulo); Hospital Tacuarembo (F Hitta); Médica Uruguaya Corporación de Asistencia Médica (P Nadales); Sanatorio Americano - Femi (H Albornoz) *Venezuela:* Hospital Dr Luis Ortega (Z Salmen); Hospital Unversitario de Caracas (C Pacheco) *Vietnam:* Bach Mai Hospital (T Bui); Franco Vietnamese Hospital (F Potie); Ninh Thuan Hospital (C Nguyen Huu)

## Electronic supplementary material

Additional file 1: Table S1: Sites of infection. **Table S2.** Antibiotic use in patients with abdominal infections. **Table S3.** Microbiology and antibiotic use in survivors and non-survivors. (DOC 117 KB)

## Abbreviations

ICU: Intensive care unit
EPIC: Extended Prevalence of Infection in the ICU
SAPS: Simplified acute physiology score
SOFA: Sequential organ failure assessment
ISF: International Sepsis Forum
SD: Standard deviation
IQR: Interquartile range
LOS: Length of stay
SOAP: SEPSIS Occurrence in Acutely Ill Patients.
