# Supplementary material for: Abdominal infections in the intensive care unit: characteristics, treatment and determinants of outcome
Source: BMC Infect Dis. 2014 Jul 29;14:420. doi: 10.1186/1471-2334-14-420 (PMC4122779; doi:10.1186/1471-2334-14-420)
Supplement: Supplementary file 1 — Additional file 1: Table S1: Sites of infection. Table S2. Antibiotic use in patients with abdominal infections. Table S3. Microbiology and antibiotic use in survivors and non-survivors. (DOC 117 KB) [file 12879_2014_3717_MOESM1_ESM.doc]

**Abdominal infections in the ICU: characteristics, treatment and determinants of outcome**

Jan De Waele MD PhD^1^, Jeffrey Lipman MD^2^, Yasser Sakr MD PhD^3^,

John C Marshall MD^4^**,** Philippe Vanhems MD^5^, Casiano Barrera Groba FFICM^6^, Marc Leone MD PhD^7^, Jean-Louis Vincent^8^ for the EPIC II Investigators

Supplemental file

**Supplemental Table 1.** Sites of infection

| **Site of infection** | **n (%)** |
| --- | --- |
| Abdomen | 1392 (100) |
| Respiratory tract | 373 (26.8) |
| Blood stream | 161 (11.6) |
| Renal/urinary tract | 93 (6.7) |
| Skin | 56 (4.0) |
| Catheter | 53 (3.8) |
| Central nervous system | 1 (0.1) |
| Other | 36 (2.6) |

**Supplemental Table 2.** Antibiotic use in patients with abdominal infections

|  | n (%) |
| --- | --- |
| **Cephalosporins** | **313 (22.5)** |
| - Cefuroxime | 55 (3.9) |
| - Cefepime OR cefpirome | 51 (3.7) |
| - Ceftazidime | 45 (3.2) |
| - Cefazoline | 19 (1.4) |
| - Other cephalosporins | 143 (10.3) |
| **Penicillins** | **537 (38.6)** |
| - Piperacillin + tazobactam | 367 (26.4) |
| - Amoxicillin + clavulanic acid | 61 (4.4) |
| - Ampicillin | 44 (3.2) |
| - Other penicillins | 46 (3.3) |
| - Benzylpenicillin | 11 (0.8) |
| - Oxacillin OR cloxacillin OR flucloxacillin | 8 (0.6) |
| **Other beta-lactam antibiotics** | **478 (34.3)** |
| - Imipenem OR meropenem | 449 (32.3) |
| - Other beta-lactams | 15 (1.1) |
| - Aztreonam | 12 (0.9) |
| - Temocillin | 2 (0.1) |
| **Aminoglycosides** | **217 (15.6)** |
| - Amikacin | 105 (7.5) |
| - Other aminoglycosides | 82 (5.9) |
| - Tobramycin | 30 (2.2) |
| **Quinolones** | **217 (15.6)** |
| - Ciprofloxacin | 159 (11.4) |
| - Other quinolones | 58 (4.2) |
| **Glycopeptides** | **347 (24.9)** |
| - Vancomycin | 296 (21.3) |
| - Other glycopeptides | 51 (3.7) |
| **Macrolides** | **32 (2.3)** |
| - Erythromycin | 6 (0.4) |
| - Other macrolides | 26 (1.8) |
| **Other antibiotics** | **672 (48.3)** |
| - Metronidazole | 459 (33.0) |
| - Oxazolidinone | 62 (4.4) |
| - Tigecycline | 34 (2.4) |
| - Cotrimoxazole | 23 (1.6) |
| - Lipopeptide | 2 (0.1) |
| - Other antibiotics | 92 (6.6) |
| **Antifungal** | **410 (29.4)** |
| - Fluconazole | 268 (19.2) |
| - Caspofungin | 60 (4.3) |
| - Amphotericin B | 38 (2.7) |
| - Voriconazole | 22 (1.6) |
| - Amphotericin B lipid formulations | 13 (0.9) |
| - Other antifungals | 9 (0.6) |
| **Antiviral** | **15 (1.1)** |

**Supplemental Table 3.** Microbiology and antibiotic use in survivors and non-survivors.

|  | **Survivors (n=917)** | **Non-survivors (n=382)** | **P** |
| --- | --- | --- | --- |
| **Microorganisms: Positive isolates** | 599 (65.3) | 267 (69.9) | 0.11 |
| **Gram-positive bacteria** | | | |
| Methicillin-resistant *Staphylococcus aureus* | 23 (3.8) | 11 (4.1) | 0.84 |
| Methicillin-sensitive *S. aureus* | 16 (2.7) | 3 (1.1) | 0.15 |
| Methicillin-sensitive coagulase-negative staphylococci | 19 (3.2) | 6 (2.2) | 0.45 |
| Methicillin-resistant coagulase-negative staphylococci | 14 (2.3) | 7 (2.6) | 0.80 |
| Enterococci, ampicillin sensitive | 78 (13.0) | 40 (15.0 | 0.44 |
| Enterococci, ampicillin resistant | 46 (7.7) | 21 (7.9) | 0.92 |
| Group A, B, C, G *Streptococcus* | 10 (1.7) | 4 (1.5) | 0.85 |
| *Streptococcus pneumoniae* | 5 (0.8) | 0 (0.0) | 0.13 |
| *Streptococcus*, other than group A, B, C and D | 20 (3.3) | 8 (3) | 0.79 |
| Gram-positive cocci, other | 4 (0.7) | 4 (1.5) | 0.24 |
| Gram-positive bacilli, other | 7 (1.2) | 1 (0.4) | 0.26 |
| **Gram-negative bacteria** | | | |
| *Escherichia coli* | 145 (24.2) | 51 (19.1) | 0.10 |
| *Enterobacter* spp. | 49 (8.2) | 20 (7.5) | 0.73 |
| *Klebsiella* spp. | 55 (9.2) | 21 (7.9) | 0.53 |
| *Proteus* spp. | 36 (6.0) | 8 (3.0) | 0.06 |
| *Salmonella* spp. | 5 (0.8) | 2 (0.7) | 0.90 |
| *Serratia* spp. | 4 (0.7) | 2 (0.7) | 0.89 |
| *Citrobacter* spp. | 7 (1.2) | 5 (1.9) | 0.41 |
| *Pseudomonas aeruginosa* | 45 (7.5) | 39 (14.6) | **0.001** |
| *Pseudomonas*, other than *P aeruginosa* | 1 (0.2) | 3 (1.1) | 0.06 |
| *Stenotrophomonas maltophilia* | 6 (1.0) | 10 (3.7) | **<0.01** |
| *Acinetobacter* spp. | 20 (3.3) | 13 (4.9) | 0.28 |
| *Campylobacter* spp. | 7 (1.2) | 0 (0.0) | 0.08 |
| *Haemophilus* spp. | 1 (0.2) | 1 (0.4) | 0.56 |
| Enterobacteria, other | 7 (1.2) | 1 (0.4) | 0.26 |
| *Bacillus* | 6 (1.0) | 5 (1.9) | 0.29 |
| **Anaerobes** | | | |
| *Clostridium* | 62 (10.4) | 28 (10.5) | 0.95 |
| Anaerobic cocci | 5 (0.8) | 2 (0.7) | 0.90 |
| *Bacteroides* | 20 (3.3) | 6 (2.2) | 0.38 |
| Anaerobes, other | 14 (2.3) | 1 (0.4) | 0.04 |
| **Fungi** | | | |
| *Candida albicans* | 79 (13.2) | 37 (13.9) | 0.79 |
| *Candida* non-albicans | 24 (4.0) | 13 (4.9) | 0.56 |
| Aspergillus | 0 (0.0) | 1 (0.4) | 0.13 |
| Fungi, other | 3 (0.5) | 2 (0.7) | 0.66 |
| **Antibiotics use** |  |  |  |
| Cephalosporins | 178 (19.4) | 61 (16.0) | 0.14 |
| Penicillins | 319 (34.8) | 134 (35.1) | 0.92 |
| Other beta-lactams | 278 (30.3) | 135 (35.3) | 0.08 |
| Aminoglycosides | 116 (12.6) | 48 (12.6) | 0.97 |
| Quinolones | 131 (14.3) | 57 (14.9) | 0.77 |
| Glycopeptides | 195 (21.3) | 118 (30.9) | <0.001 |
| Macrolides | 20 (2.2) | 8 (2.1) | 0.92 |
| Other antibiotics | 360 (39.3) | 157 (41.1) | 0.54 |
| Antifungals | 210 (22.9) | 115 (30.1) | <0.01 |
